# Allylation of *C*‐, *N*‐, and *O*‐Nucleophiles via a Mechanochemically‐Driven Tsuji–Trost Reaction Suitable for Late‐Stage Modification of Bioactive Molecules

**DOI:** 10.1002/ange.202314637

**Published:** 2023-11-29

**Authors:** Johanna Templ, Michael Schnürch

**Affiliations:** ^1^ Institute of Applied Synthetic Chemistry, TU Wien Getreidemarkt 9/E163 1060 Vienna Austria

**Keywords:** Ammonium Salts, Ball Milling, Green Chemistry, Mechanochemistry, Solvent Free

## Abstract

We present the first solvent‐free, mechanochemical protocol for a palladium‐catalyzed Tsuji–Trost allylation. This approach features exceptionally low catalyst loadings (0.5 mol %), short reaction times (<90 min), and a simple setup, eliminating the need for air or moisture precautions, making the process highly efficient and environmentally benign. We introduce solid, nontoxic, and easy‐to‐handle allyl trimethylammonium salts as valuable alternative to volatile or hazardous reagents. Our approach enables the allylation of various *O*‐, *N*‐, and *C*‐nucleophiles in yields up to 99 % even for structurally complex bioactive compounds, owing to its mild conditions and exceptional functional group tolerance.

## Graphical abstract

A mechanochemical, solvent‐free protocol for Tsuji–Trost allylation of *O*‐, *N*‐, and *C*‐Nucleophiles using nontoxic, solid allyl trimethylammonium chloride as alternative allylating agent is presented. This method features fast reaction kinetics, very low catalyst loadings, high yields, mild conditions, and high functional group tolerance. Its potential for late‐stage modifications of bioactive compounds is demonstrated through multiple examples.

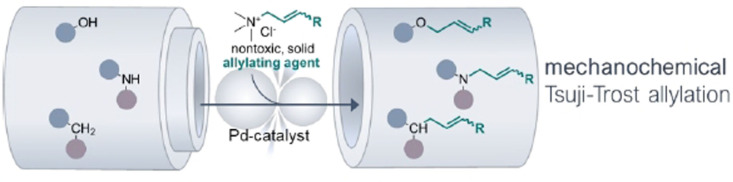

In recent years, mechanochemistry has emerged as a revolutionary tool in modern synthetic chemistry.^[1]^ Especially en route to more sustainable and environmentally benign chemical processes, mechanochemical transformations are privileged,^[2]^ since this approach offers a unique advantage: the ability to conduct conventional synthetic reactions without the need for solvents coupled with rapid reaction kinetics. These unique features make mechanochemistry highly attractive for both industrial applications^[3]^ and fundamental research across diverse fields, including material science, inorganic chemistry, and organic chemistry.[^1a^, ^2c^, ^2d^, ^4^]

In the past few years, significant efforts have been made to devising more economical protocols for palladium‐catalyzed reactions employing ball milling setups that rely solely on mechanical force for energy input.^[5]^ To date, mechanochemical adaptions of various fundamental Pd‐catalyzed reactions, such as the Negishi,^[6]^ Mizoroki–Heck,^[7]^ Suzuki–Miyaura,^[8]^ Buchwald–Hartwig,^[9]^ Sonogashira,^[10]^ a C−S coupling^[11]^ and C−H arylation^[12]^ have been successfully established. To further enrich this “green” toolbox, we present a novel and solvent‐free protocol for a mechanochemical palladium‐catalyzed Tsuji–Trost allylation of *O‐*, *N‐*, and *C‐*nucleophiles using a ball mill reactor (Figure 1).

**Figure 1:**
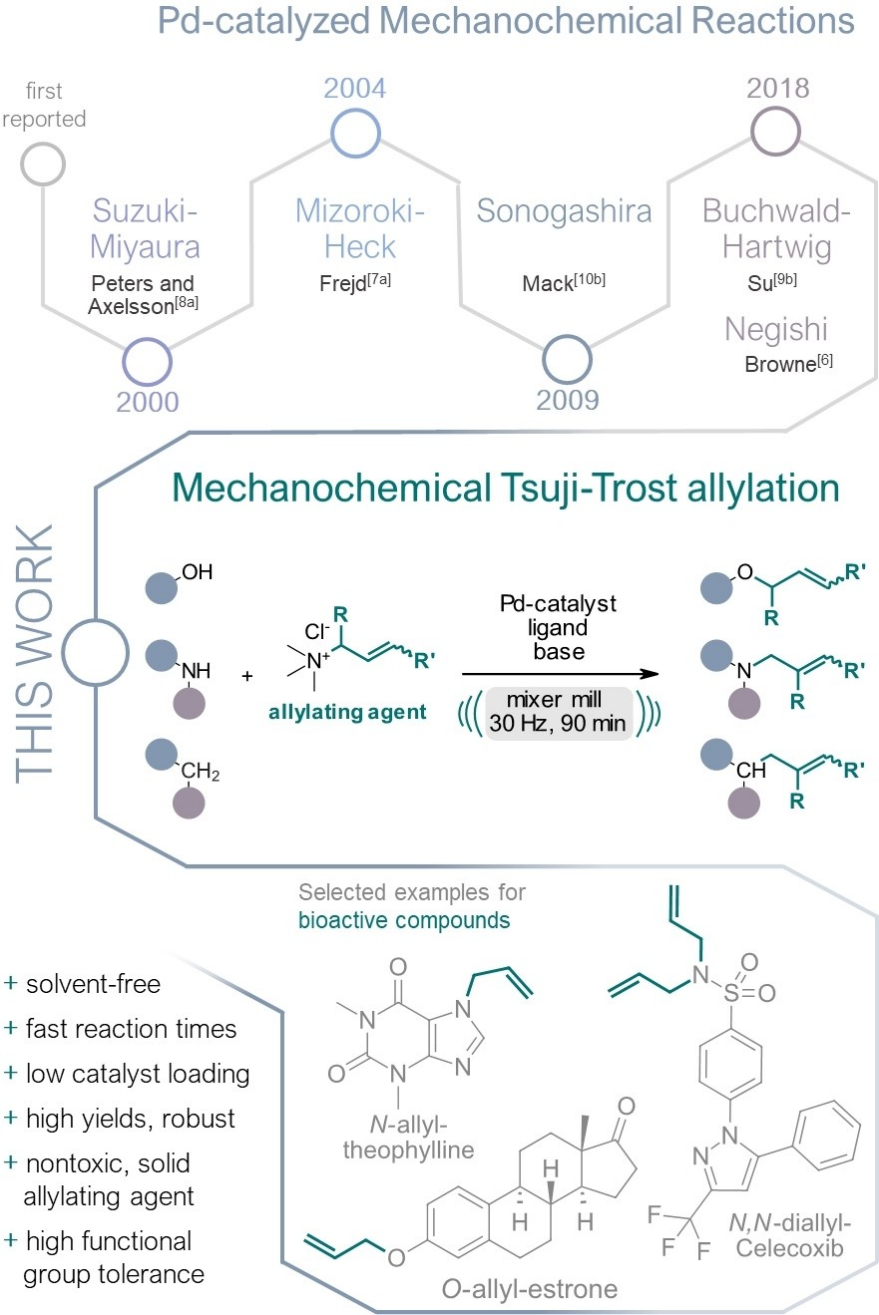
Pd‐catalyzed mechanochemical reactions according to their first appearance in literature (top) and the mechanochemical Tsuji–Trost allylation (middle) with an outline of potential application in late‐stage functionalization of bioactive compounds (bottom).

Our endeavor prioritized the development of an environmentally benign and entirely safe process, aligning with the principles of green chemistry.

In the Tsuji–Trost reaction, the presence of a leaving group in the allylic position of the substrate is crucial.^[13]^ Numerous protocols have been developed for various leaving groups, encompassing halides, carbonates, acetates, hydroxy groups, and amides.^[14]^ Notably, quaternary ammonium moieties in allylic positions are seldomly employed as leaving groups with only limited examples documented in literature.^[15]^ Nevertheless, harnessing these quaternary ammonium salts as the allyl source presents a distinctive advantage, especially when considering the potential hazards and toxicity associated with traditionally applied allylating agents, such as allyl chloride or bromide. Quaternary ammonium salts are nontoxic and pose significantly fewer health and safety risks. Moreover, their solid nature drastically simplifies the handling. Consequently, they represent a valuable and sustainable alternative to hazardous reagents.^[16]^

The allylic trimethylammonium chlorides employed in this protocol solely release gaseous trimethylamine as the leaving group, obviating the need for an additional byproduct separation step. This feature can be particularly advantageous in the pharmaceutical industry^[17]^ or when the product is intended for use in subsequent reactions without further purification.

Building upon our prior research involving ammonium salts as solid alkylating agents,[^16^, ^18^] we initiated our current investigation using allyl trimethylammonium chloride as the allylating agent (**II**), paired with 4‐hydroxybiphenyl (**I**) serving as the nucleophile. Based on mechanistic considerations^[19]^ of the catalytic cycle for Tsuji–Trost allylation, where a leaving group elimination prompts the formation of an η_3_ π‐allyl‐Pd^II^ complex, we commenced our optimization studies (see Figure 2) by selecting a known and robust catalytic system of [Pd(allyl)Cl]_2_ (**cat** **1**) with cheap *rac*‐[2,2′‐bis(diphenylphosphino)‐1,1′‐binaphthyl] (*rac*‐BINAP) ligand (**L1**).^[20]^ Initially, 30 mol % of Cs_2_CO_3_ were added, aligning with precedent literature recommendations[^20^, ^21^] and the allylated product was obtained in 42 % yield (entry A2). Increasing the amount of base to 1.1 equiv. drastically increased the yield of **1** to 77 % and even 93 % when 2 equiv. were used. As no significant difference in yields could be observed when using Cs_2_CO_3_ or K_2_CO_3_ as the base, we continued our studies with the latter cheaper base (cf. entry A4 and A5). Through systematic optimization, we achieved a remarkable reduction of catalyst and ligand loadings to as little as 0.5 mol % and 1 mol % (entry B4), respectively, and reduction of the milling time to as short as 90 min, while still attaining an excellent yield of 97 % (entry C3). Excluding both the catalyst and the ligand (entry B5) or the ligand only (entry B6), no or only 5 % product formation could be observed. These findings prove that the reaction requires the catalytic system to operate, distinctively from a conventional nucleophilic substitution mechanism. Finally, we conducted a reaction in toluene (0.5 M) at 60 °C with a comparable reaction time of 90 min (entry C5). Only 19 % product formation could be observed, underscoring the typically accelerated reaction kinetics inherent to mechanochemical reactions.^[2b]^

**Figure 2:**
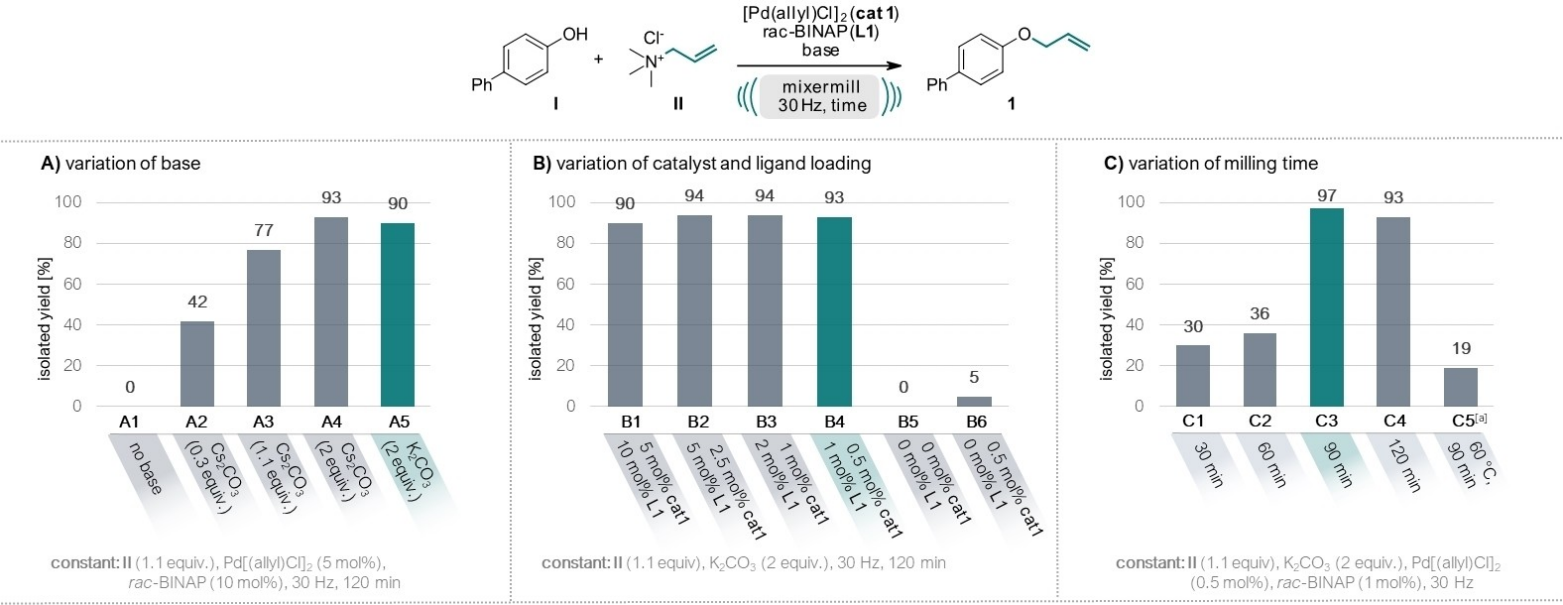
Optimization of the reaction conditions for the mechanochemical Tsuji–Trost allylation. Reactions were performed on a 0.5 mmol scale under air in an IST636 mixer mill, using a Teflon milling jar (7 mL) and two ZrO_2_ milling balls (one with 7 mm diameter, one with 10 mm diameter, see Supporting Information for details) at a frequency of 30 Hz. Allyl trimethylammonium chloride (**II**) was used as allylating agent and 4‐hydroxybiphenyl (**I**) as nucleophile, [Pd(allyl)Cl]_2_ as catalyst (**cat** **1**) and *rac*‐BINAP as ligand (**L** **1**). Full table see SI. [a] Reaction performed in toluene (0.5 M) at 60 °C with a reaction time of 90 min.

We initiated our scope evaluation by testing various nucleophiles (Scheme 1), including *O‐*, *N‐*, and *C‐*nucleophiles, with commercially available allyl trimethylammonium chloride (**II**) under the optimized reaction conditions (Figure 2, entry C3). The choice between potassium or cesium carbonate as the base depended on the specific reaction‘s yield. For most substrates, pure allylated product was obtained solely through silica filtration (for further details see the SI). Phenol nucleophiles consistently delivered excellent yields ranging from 61 % to 99 % (**1**–**13**, **17**, **18**, and **21**) independent of their substitution pattern. Electron‐donating (**1**, **2**, **8**–**11**, and **17**) and electron‐withdrawing (**4**–**6**, **12**, **13**) groups as well as combinations thereof (**5** and **7**), were tolerated at various positions on the aryl ring. Aliphatic (**15**, **16**, and **20**) and allylic (**19**) alcohols also readily underwent allylation, with yields reaching 96 %. Even the complex bioactive compound estrone was quantitatively allylated (**21**). To underscore the robustness and practicality of this approach, we conducted the synthesis of product **1** on a 1 g scale. The reaction scale‐up involved an increase of the dimensions of the milling vessels and the milling balls, yet the reaction yielded comparable results, with silica filtration sufficing to obtain pure product **1** in a quantitative yield of 1.15 g.

**Scheme 1:**
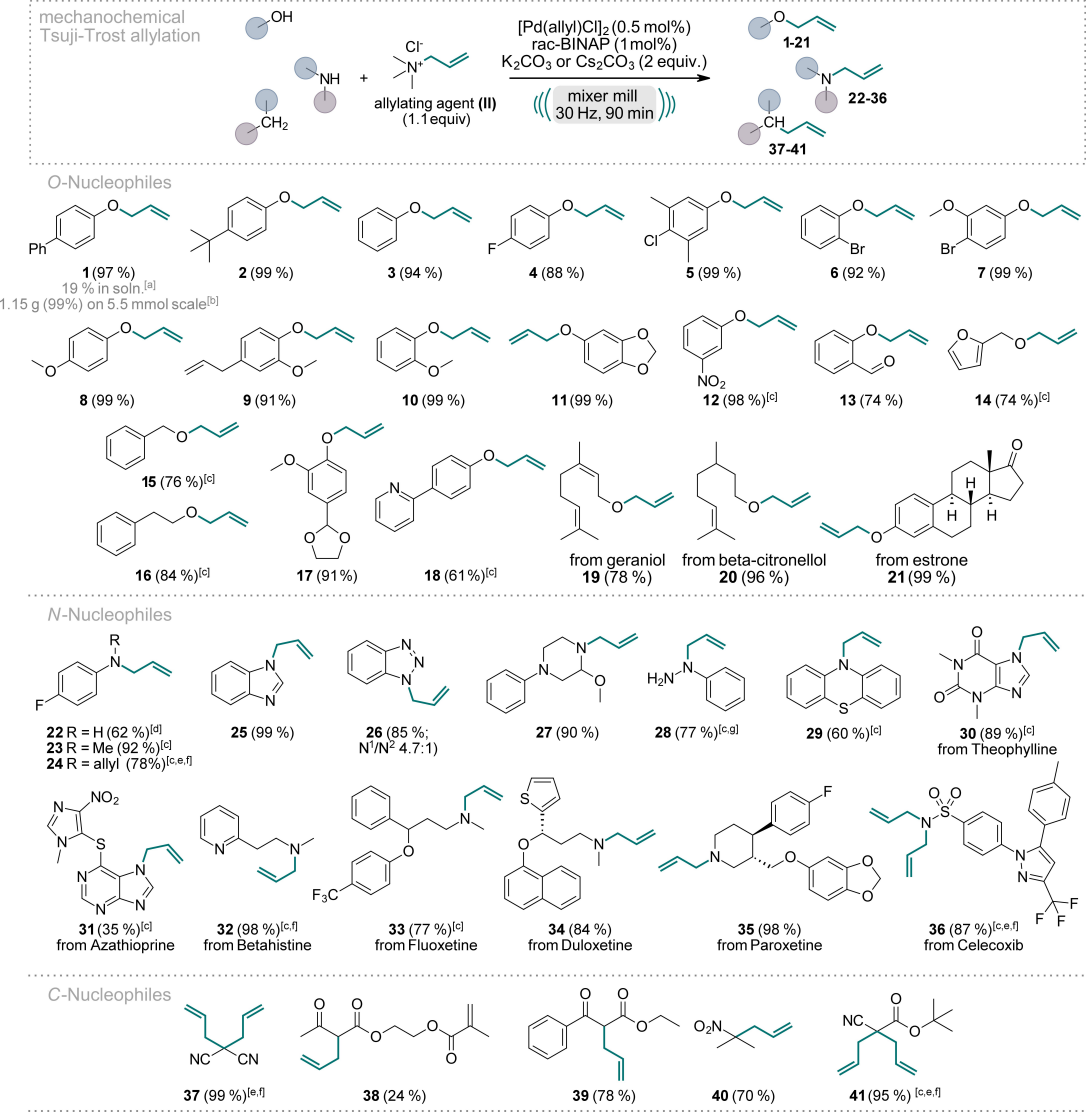
Scope of the Pd‐catalyzed, mechanochemical Tsuji–Trost reaction of *O‐*, *N‐*, and *C‐*nucleophiles. Reactions were performed on a 0.5 mmol scale under air in an IST636 mixer mill, using a Teflon milling jar (7 mL) and two ZrO_2_ milling balls (one with 7 mm diameter, one with 10 mm diameter, see Supporting Information for details) with a milling time of 90 minutes at a frequency of 30 Hz. If not stated otherwise, K_2_CO_3_ was used as the base. Isolated yields are shown. [a] Reaction performed in toluene (0.5 M) at 60 °C with a reaction time of 90 min. [b] Reaction was performed on a 5.5 mmol scale using 2 Teflon milling jars (25 mL) each equipped with two 12.7 mm ZrO_2_ milling balls. [c] Cs_2_CO_3_ was used as the base. [d] No base. [e] 2.5 equiv. of **II**. [f] 3 equiv. of the base were used. [g] 1 equiv. of **II**, 1 mol % Pd[(allyl)Cl]_2_, 2 mol % *rac*‐BINAP.

Given the significance of nitrogen‐containing motifs in biologically active compounds, we next focused on *N‐*nucleophiles, particularly in small molecule drugs and frequently encountered motifs thereof.^[22]^ Aniline derivatives could be mono‐ or bis‐allylated (**22**–**24**). However, primary amines showed diminished yields due to the challenging prevention of over‐alkylation (**22**). Secondary amines, on the other hand, gave excellent allylation yields without overalkylation concerns (**23**, **27**, **32**–**35**).

Nitrogen containing heterocycles are important motifs due to their presence in numerous biologically active compounds. Gratifyingly, our protocol allowed efficient allylation of many *N‐*heterocyclic systems, both aromatic and aliphatic ones. Benzotriazole gave a high overall yield of 85 % (**26**), with the allylation occurring predominantly at the *N* ^*1*^‐position, and to some extent at position *N* ^*2*^ at a 4.75 : 1 ratio. Notably, column chromatography easily separated these two isomers. Other *N‐*heterocyclic compounds were allylated with excellent yields, including benzimidazole towards product **25** and Theophylline giving product **30**. Unfortunately, bioactive Azathioprine could be allylated only in a modest yield of 35 % (**31**), likely due to its steric congestion. Phenylhydrazine was exclusively allylated at the *N* ^*1*^‐position, consistent with recent literature reports^[23]^ (product **28**). The sulfonamide moiety in Celecoxib was fully bis‐allylated as its high nucleophilicity hampers a monoselective reaction (**36**). This mild protocol successfully facilitated the allylation of several complex *N‐*containing small molecule drugs (**30**–**36**), highlighting its potential in late‐stage modifications.

*C‐*nucleophiles bearing electron‐withdrawing groups in alpha‐position could be mono‐allylated, giving products **38**–**40**, or fully converted to the bis‐allylated products (**37** and **41**) with yields up to 99 %. Notably, in the case of product **39**, the competitive byproduct formed via *O‐*allylation of the enolate was significantly reduced when K_2_CO_3_ was employed as the base, rather than Cs_2_CO_3_.

Subsequently, we explored the versatility of substituted allyl ammonium salts (Scheme 2) with *O‐*, *N‐*, and *C‐*nucleophiles (**42**–**51**). These salts can be readily derived from the respective allyl chlorides by reacting with Me_3_N solution. Initially, when employing the freshly synthesized and recrystallized ammonium salt from cinnamyl chloride, the yield of product **44** did not exceed 20 %. However, as others have calculated, we made the noteworthy discovery that the addition of water significantly accelerated the reaction.^[24]^ Specifically, when we conducted the reaction with 4‐hydroxybiphenyl as the nucleophile and dried cinnamyl trimethylammonium chloride as the allylating agent, only 6 % of product **44** was obtained when additional molecular sieves were added to the milling vessel. In contrast, when 2 equivalents of water were added, the yield of **44** increased to 63 %, and upon the addition of 5 equivalents of water, the reaction proceeded with complete conversion, and an isolated yield of 96 %. This water‐mediated enhancement of reaction rates was consistently observed with all other synthesized allyl ammonium salts employed in this protocol. Alternatively, the acceleration of the reaction by addition of water might be due to the effect of liquid assisted grinding,^[25]^ or a combination of both factors. Interestingly, when employing terminal allyl ammonium compounds, exclusively linear products were obtained with moderate to excellent yields (**44**–**46**, **49**–**51**). To ensure a cleaner reaction, we slightly increased the catalyst loading to 1.5 mol % since it resulted in the exclusive formation of the trans‐isomer. Due to competing bis‐allylation, yields for the *C‐*allylated products **50** and **51** were diminished. Interestingly, the 3,3‐dimethylallyl trimethylammonium chloride did not give the desired product **52** but only starting material was recovered, assumingly due to steric hindrance upon Pd‐allyl complex formation and subsequent nucleophilic attack.

**Scheme 2:**
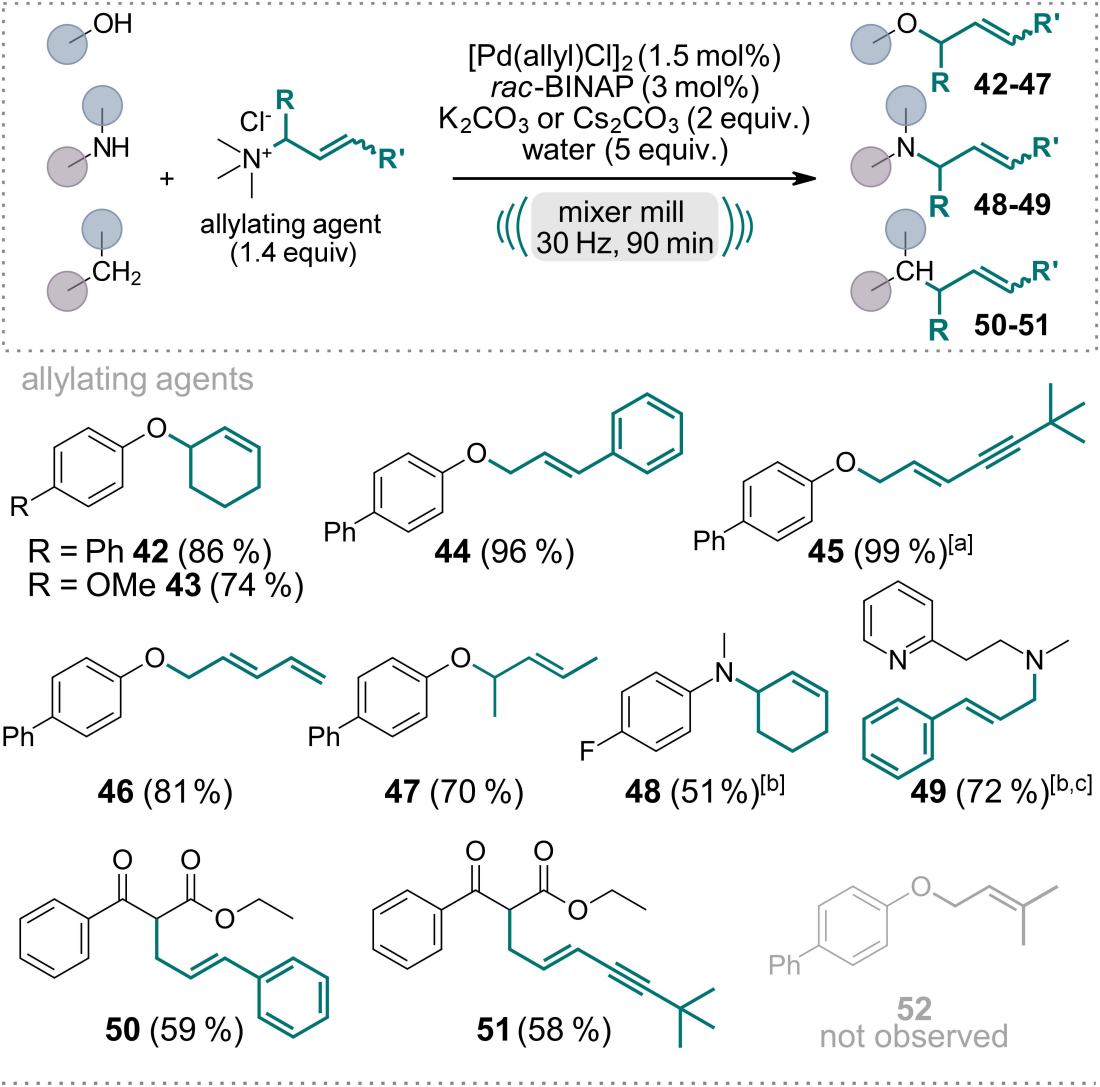
Scope of the Pd‐catalyzed, mechanochemical Tsuji–Trost reaction employing various substituted allyl trimethylammonium salts as allylating agents. Reactions were performed on a 0.5 mmol scale under air in an IST636 mixer mill, using a Teflon milling jar (7 mL) and two ZrO_2_ milling balls (one with 7 mm diameter, one with 10 mm diameter, see Supporting Information for details) with a milling time of 90 minutes at a frequency of 30 Hz. If not stated otherwise, K_2_CO_3_ was used as the base. Isolated yields are shown. [a] 2 mol % of Pd[(allyl)Cl]_2_ and 4 mol % *rac*‐BINAP were used. [b] Cs_2_CO_3_ was used as the base. [c] 3 equiv. of base were used.

This novel reaction, employing allyl trimethylammonium chloride as the allylating agent, achieves an exceptional level of selectivity, yielding only a single product. This is in stark contrast to using the corresponding allyl chloride directly under the conditions outlined. For example, using cinnamyl chloride instead of cinnamyl trimethylammonium chloride under the reaction conditions described in Scheme 2, the crude NMR reveals a complex mixture of products and starting material. Conversely, when alternative cinnamyl trimethylammonium chloride is utilized, the reaction yields pure product **44** after simple silica filtration.

Prompted by our results, we explored the potential for enantioselective reactions using chiral ligands (Scheme 3, for further details see SI). Several enantioselective reactions using ball milling conditions are already established under organocatalytic conditions.^[26]^ In contrast, metal‐catalyzed mechanochemical reactions have received only limited attention so far. Employing a chiral (*R*)‐BINAP ligand, we achieved an enantiomeric excess (***ee***) of 28 % under the described conditions. Gratifyingly, using a chiral (*R*)‐5,5′‐bi(diphenylphosphino)‐4,4′‐bi‐1,3‐benzodioxole ((*R*)‐SEGPHOS) ligand, we obtained compound **(*R*)** *‐* **43** in a yield of 73 % with a moderate ***ee*** of 52 %. Further investigations to improve the *ee*‐values are currently underway in our laboratories.

**Scheme 3:**
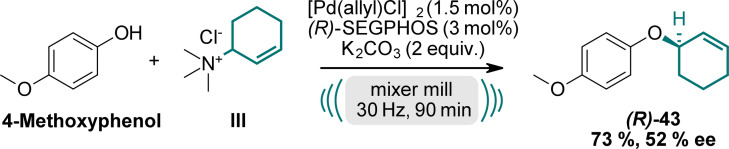
Enantioselective reaction towards **(*R*)** *‐* **43** using chiral (*R*)‐SEGPHOS ligand.

In summary, we have established a solvent‐free, robust, and high‐yielding protocol for a palladium‐catalyzed mechanochemical Tsuji–Trost allylation. This method offers an easy setup without the need for air or moisture precautions, very low catalyst and ligand loading, very mild reaction conditions, short reaction times, and high conversion rates. The outstanding functional group tolerance allows clean and mild late‐stage allylation of complex bioactive compounds. This study underscores the underappreciated potential of solid, nontoxic allyl ammonium salts as allylating agents in Tsuji–Trost reactions, offering a valuable and environmentally friendly alternative to traditional hazardous allylation reactions.

## Supporting Information

Complete optimization screening data, experimental procedures, and characterization data for all compounds isolated.

The authors have cited additional references within the Supporting Information.[^20^, ^27^, ^28^, ^29^, ^30^, ^31^, ^32^, ^33^, ^34^, ^35^, ^36^, ^37^, ^38^, ^39^, ^40^, ^41^, ^42^, ^43^, ^44^, ^45^, ^46^, ^47^, ^48^, ^49^, ^50^, ^51^, ^52^, ^53^, ^54^, ^55^, ^56^]

## Conflict of interest

The authors declare no conflict of interest.

## Supporting information

As a service to our authors and readers, this journal provides supporting information supplied by the authors. Such materials are peer reviewed and may be re‐organized for online delivery, but are not copy‐edited or typeset. Technical support issues arising from supporting information (other than missing files) should be addressed to the authors.

Supporting Information

## Data Availability

The data that support the findings of this study are available in the supplementary material of this article.
